# Variation in Gene Expression between Two *Sorghum bicolor* Lines Differing in Innate Immunity Response

**DOI:** 10.3390/plants10081536

**Published:** 2021-07-27

**Authors:** Yaya Cui, Dongqin Chen, Yuexu Jiang, Dong Xu, Peter Balint-Kurti, Gary Stacey

**Affiliations:** 1 Divisions of Plant Science and Technology and Biochemistry, C. S. Bond Life Sciences Center, University of Missouri, Columbia, MO 65211, USA; cuiy@missouri.edu (Y.C.); chendq@cau.edu.cn (D.C.); 2 Department of Electrical Engineering and Computer Science, C. S. Bond Life Sciences Center, University of Missouri, Columbia, MO 65211, USA; yjm85@mail.missouri.edu (Y.J.); xudong@missouri.edu (D.X.); 3 Department of Entomology and Plant Pathology, NC State University, Raleigh, NC 27695, USA; pjbalint@ncsu.edu; 4 Plant Science Research Unit, USDA-ARS, Raleigh, NC 27695, USA

**Keywords:** *Sorghum bicolor*, ROS, innate immunity, MAMPs, RNA-seq, gene expression

## Abstract

Microbe associated molecular pattern (MAMPs) triggered immunity (MTI) is a key component of the plant innate immunity response to microbial recognition. However, most of our current knowledge of MTI comes from model plants (i.e., *Arabidopsis thaliana*) with comparatively less work done using crop plants. In this work, we studied the MAMP triggered oxidative burst (ROS) and the transcriptional response in two *Sorghum bicolor* genotypes, BTx623 and SC155-14E. SC155-14E is a line that shows high anthracnose resistance and the line BTx623 is susceptible to anthracnose. Our results revealed a clear variation in gene expression and ROS in response to either flagellin (flg22) or chitin elicitation between the two lines. While the transcriptional response to each MAMP and in each line was unique there was a considerable degree of overlap, and we were able to define a core set of genes associated with the sorghum MAMP transcriptional response. The GO term and KEGG pathway enrichment analysis discovered more immunity and pathogen resistance related DEGs in MAMP treated SC155-14E samples than in BTx623 with the same treatment. The results provide a baseline for future studies to investigate innate immunity pathways in sorghum, including efforts to enhance disease resistance.

## 1. Introduction

Plants are naturally exposed to a variety of stresses, including numerous bacterial, fungal, and viral pathogens [1]. Plants protect themselves from pathogens using pre-formed structures and chemicals, and infection-induced responses of the immune system. The plant immune system employs a two-tiered perception system with two interconnected layers of receptors. The first tier is activated by surface-localized pattern recognition receptors (PRRs), which sense molecules outside the cell by recognizing the invading pathogen through detection of conserved structural motifs, termed Microbe-Associated Molecular Patterns (MAMPs), which trigger a moderate, but broad, defense response [2]. Flagellin, chitin, lipopolysaccharides, peptidoglycans (PGNs), and elongation factor Tu are examples of the well-studied MAMPs [3,4,5]. This defense response is termed MAMP-trigged immunity (MTI) [6]. The second tier of defense known as the Effector-Triggered Immunity (ETI) occurs in response to specific pathogen-derived effector proteins that are recognized by the plant through the action of resistance proteins, resulting in a strong, but highly specific immune response. ETI can often be recognized by the induction of localized cell death termed a hypersensitive response (HR) [7]. Both MTI and ETI systems sense the pathogens and respond by activating antimicrobial defenses in the infected cell and neighboring cells [3,8]. Plants also carry immune receptors that recognize highly variable pathogen effectors, these include the NBS-LRR class of proteins [9]. In addition, systemic acquired resistance (SAR) is a “whole-plant” resistance response that occurs following an earlier localized exposure to a pathogen. SAR is associated with the induction of a wide range of genes (pathogenesis-related genes), and the activation of SAR requires the accumulation of endogenous salicylic acid (SA) [10].

Studies primarily done using *Arabidopsis* have shown that MTI can cause a variety of responses including changes in reactive oxygen species production (ROS), reactive nitrogen species production such as nitric oxide (NO), callose deposition, intracellular calcium levels, ion flux across the plasma membrane, induction or repression of the expression of several plant defense related genes, alterations in the plant cell wall, induction of antimicrobial compounds and the synthesis of pathogenesis-related (PR) proteins [3,11]. Although several of these MTI responses can be considered general plant responses, their magnitude can be plant-species specific and even cultivar specific [12,13,14,15,16,17]. Methods have been developed to quantify the plant MTI response. These methods include measuring ROS or NO production, mitogen-activated protein kinase phosphorylation, specific MAMP-induced gene expression, seedling growth inhibition, lignin and cell wall-bound phenols, and MAMP-induced resistance to bacterial and fungal pathogens [18,19,20].

*Sorghum bicolor* (L.) Moench is a key, global cereal crop that has been adapted to a range of habitats and bred for diverse purposes [21]. The cultivation of sorghum is expected to increase substantially worldwide as one of the major crops for food and biomass production [22]. As the practices and geographical areas under sorghum cultivation increase, it is inevitable that the disease pressure will increase. Sorghum is distinguished among other cereals by its unusually broad range of diseases, which is one of the most important constraints to its production. Globally, widespread fungal diseases are the most destructive sorghum diseases that result in huge losses in yield, both in terms of the quantity and quality of the grains [23]. Anthracnose is one of the most severe fungal diseases affecting sorghum grain yield and biomass production. Yield losses due to this pathogen can be 50 to 70% in susceptible cultivars [24,25]. Plants have developed various defense strategies to fight against pathogen invasion. In sorghum, these strategies include activation of PR proteins [26], accumulation of hydrogen peroxide [27], and biosynthesis of flavonoid phytoalexins [28]. Different approaches have been used to study sorghum’s resistance responses, identify defense compounds, and identify physical barriers against anthracnose (see reference [29,30] for review). Gene expression studies were widely used to identify candidate resistance genes in plants based on the differential expression between resistant and susceptible cultivars or non-inoculated and pathogen inoculated plants (e.g., references [31,32,33,34,35,36,37]). Wang et al., [37] performed transcriptomic analysis to study the response of sorghum cultivar BTx623 to *C. sublineola*. They reported that genes involved in phenylpropanoid metabolism, and the biosynthesis of secondary metabolites were identified as differentially expressed in response to infection with the pathogen relative to non-inoculated control plants. However, these experiments were conducted with seedlings of the susceptible cultivar BTx623. Additional transcriptomic studies to compare gene expression response to a pathogen in the susceptible and resistant sorghum cultivars would be of value. Hence, it is important to understand the genetic architecture controlling sorghum basal disease resistance and its connection to quantitative disease resistance.

As an initial step in this direction, we recently screened a set of diverse sorghum germplasm for variation in their response to flg22 and chitin elicitation, as well as fungal disease resistance [38]. In this case, MTI was quantified by measuring ROS production upon MAMP elicitation. Among the outcomes of this earlier study, was the identification of sorghum genotypes that showed either a strong or weak response to MAMP elicitation.

Next generation high-throughput sequencing and study of transcriptomes can help to clarify fungal infection responses in plants and increase understanding about host responses. Transcriptome analyses were widely used to study plant responses to fungal infection in many plant species including cereal crops (e.g., references [31,32,33,34,35,36,37]). However, published data of comparative transcriptome analysis of cereal crops have primarily focused on the response to various pathogens. In sorghum, several publications focused on transcriptome comparisons of plants treated with various pathogens, including anthracnose [35,37]. Some previous publications suggested that there is no significant correlation between the response to MAMPs and fungal disease. For example, Zhang et al. [17] reported a strong correlation between response to two MAMPs flg22 and chitin in a maize mapping population but no correspondence to response to fungal disease. Kimball et al. [38] also reported a low correlation between flg22-induced ROS response scores and target leaf spot resistant scores in two sorghum RIL populations including a BTx623/SC155-14E population. Therefore, given the paucity of data, we felt it important to transcriptionally profile the sorghum response to MAMP treatments.

In this current report, we extend our earlier analysis by measuring the global transcriptional response to MAMP elicitation. We used Illumina-based RNA sequencing analysis to survey transcriptional changes in response to either flg22 or chitin treatment in the sorghum genotypes BTx623 and SC155-14E, which we previously characterized as respectively weak and strong MTI responders, respectively. BTx623 is the *Sorghum bicolor* genome reference line [22] and SC155-14E is a line showing elevated anthracnose resistance [39]. Patil et al. [39] reported that SC155-14E displayed a high level of stable resistance (nearly disease free) to anthracnose disease in all environments tested while BTx623 is susceptible to this fungal disease. Our RNA-seq analysis identified 5252 and 8085 Differentially Expressed Genes (DEGs) in BTx623 treated with flg22 and chitin, respectively, and 3849 and 5786 DEGs in flg22 and chitin treated SC155-14E, respectively, compared to untreated mock controls of these two genotypes. Furthermore, a comparison of DEGs selected using two different bioinformatic pipelines showed very similar results. Expression profiles of the differentially expressed genes, gene ontology (GO) enrichment analysis, and KEGG pathway enrichment analysis showed a clear genotype-based variation in MTI-related gene expression. The GO term and KEGG pathway enrichment results revealed more immunity and pathogen resistance related DEGs (PR genes, genes response to chitin, genes response to salicylic acid, genes response to stress, etc.) presented in MAMP treated SC155-14E samples relative to MAMP treated BTx623.The results provide a baseline for identifying the various components of the sorghum MTI response.

## 2. Results and Discussion

### 2.1. Variation of the Oxidative Burst in Different Sorghum Leaves

Our previous analysis of the MTI response of diverse sorghum genotypes revealed significant variation in the MAMP-triggered oxidative burst between the genotypes BTx623 and SC155-14E [38]. Moreover, SC155-14E is a line showing high anthracnose resistance, while line BTx623 is susceptible to anthracnose [39]. It was for these reasons, that these two genotypes were chosen for comparison using RNA-seq.

**Figure 1:**
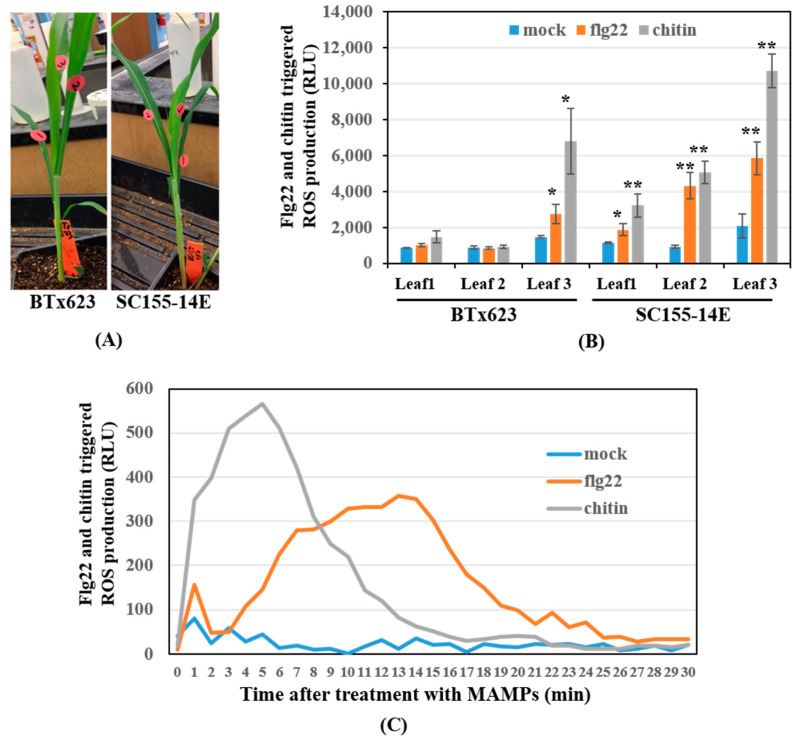
Effect of leaf position on the ROS response to MAMP elicitation in sorghum genotypes BTx623 and SC155-14E. (**A**) Fifteen-day old BTx623 and SC155-14E plants. The leaves from bottom to top were marked as leaf #1 (older leaf), leaf #2 (2nd youngest leaf) and leaf #3 (youngest leaf). (**B**) ROS response to flg22 or chitin elicitation of all three leaves of fifteen-day old BTx623 and SC155-14E plants. RLU, relative light units; Error bars indicate ± SEM; *n* = 8; * <0.05 (*t* test) and ** <0.01. (**C**) Time course of flg22-triggered and chitin-triggered ROS production in leaf # 2 of SC155-14E.

While conducting the earlier ROS measurements, we found significant variation even within the same plant, which led us to investigate the source of this variation more carefully. Specifically, we tested ROS production using three sets of leaves, going from the bottom to the top of fifteen-day-old BTx623 and SC155-14E plants. The leaves were marked as #1, #2, and #3, bottom to top as shown in Figure 1A. The ROS assay results (Figure 1B) revealed that: (1) the ROS production level was lower in the older leaves (leaf #1), while the highest levels of ROS production in response to either flg22 or chitin were from the youngest leaves (leaf #3). (2) Both MAMP-treated and untreated BTx623 2nd youngest leaves (leaf #2) produced comparably low levels of ROS production. By contrast, MAMP-treated 2nd youngest leaves of SC155-14E produced significantly (at 0.01 level) higher levels of ROS production than the untreated mock, as well as the treated or mock of BTx623 2nd youngest leaves. (3) The oxidative burst triggered by applications of flg22, or chitin was detected in leaf discs from the youngest leaves (leaf #3) of both genotypes, although, as previously observed, the levels of ROS production were higher in MAMP treated SC155-14E relative to BTx623. Time course of flg22-triggered and chitin-triggered ROS production in SC155-14E 2nd youngest leaf revealed that chitin-triggered ROS production occurred earlier than flg22-triggered ROS production (Figure 1C). Similar results were reported by Zhang et al. [17] where the chitin-triggered ROS production appeared earlier than flg22-triggered ROS production in flg22 and chitin treated maize seedlings. It was reported [40] that ROS production in response to flg22 showed the highest production of ROS around 12 min, and was the lowest at 30 min in *Arabidopsis*. Our ROS assay results in sorghum showed the highest ROS production around 13 min, and the lowest at 30 min after flg22 treatment, which is very similar to the results in *Arabidopsis*. Since our goal was to find the optimal conditions for comparison of the MTI response of the two genotypes, we chose the 2nd youngest leaves of 15-day old plants for RNA extraction and subsequent transcriptomic analysis. The variation among leaves of single sorghum plants is a reminder that results can vary between experiments depending on the source, timing, and other features of a given experiment and, hence, that experimental parameters need to be carefully controlled.

The oxidative burst, a rapid, transient, production of reactive oxygen species (ROS), is one of the earliest observable aspects of a plant’s defense response [41]. Considering that the ROS burst is an immediate response, a slightly later transcriptional response of genes related to MTI is expected. We harvested samples 60 min after MAMPs treatment for RNA-seq analysis.

### 2.2. Differentially Expressed Genes (DEGs) of BTx623 and SC155-14E in Response to MAMP Treatments

To evaluate the expression level of annotated *Sorghum bicolor* protein-coding genes for each sample, the number of clean reads of RNA-seq that mapped to each gene was calculated, and then normalized into FPKM (fragments per kb exon model per million mapped fragments). The expression changes of each gene in MAMP-treated leaf discs of BTx623 and SC155-14E in comparison to mock (water) treatments were investigated. The total expressed genes were similar in all treatments of both genotypes: 27,171, 27,283, 27,161, 27,182, 27,512 and 27,175 genes expressed in mock of BTx623, flg22-treated BTx623, chitin-treated BTx623, mock of SC155-14E, flg22-treated SC155-14E, and chitin-treated SC155-14E, respectively. We used the BTx623 reference genome for read mapping in both lines since the specific reference for SC155-14E is not available. The expressed gene numbers retrieved from BTx623 and SC155-14E with all conditions above revealed that both lines shared similar numbers of expressed genes. The overall read mapping rates of samples of SC155-14E mock (3 replicates), SC155-14E treated with flg22 (3 replicates), SC155-14E treated with chitin (3 replicates), BTx623 mock (3 replicates), BTx623 treated with flg22 (3 replicates), BTx623 treated with chitin (3 replicates) were: 93.6%, 93.9%, 93.9%, 93.8%, 93.9%, 93.6%, 93.5%, 93.5%, 93.1%, 96.9%, 97.0%, 97.0%, 97.2%, 96.8%, 97.0%, 96.9%, 97.0%, 97.0%, respectively. These results suggested that the different effects of MAMPs treatment on transcriptomes of BTx623 and SC155-14E were not due to the genomic sequence differences between the two genotypes.

**Figure 2:**
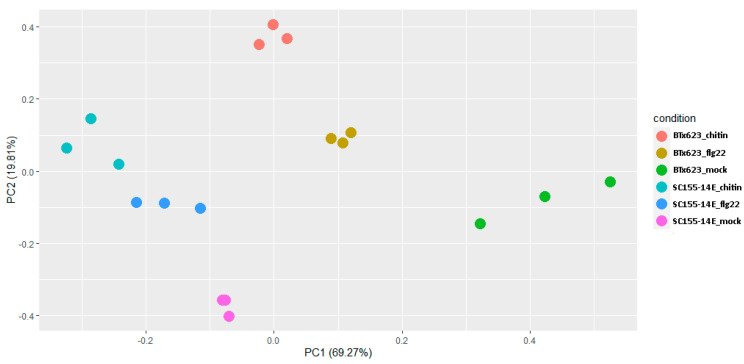
PCA plot of RNA-seq data of BTx623 and SC155-14E treated with flg22 or chitin as well as the mock-treatment. Sample plotting on the PC1 and PC2 showed the association between samples of three biological replicates of each sorghum line and each MAMP treatment. Each dot represents a condition after mapping from the original feature space (gene expression) to the first two principle components. Dots belong to the same condition tend to cluster together.

The gene expression PCA plot provides insights into the association between samples. To explore the similarity of our samples, we performed PCA analysis. Sample plotting on the PC1 and PC2 (Figure 2) showed that four experimental conditions (BTX623_flg22; SC155-14E_flg22 flg22; BTX623_chitin and SC155-14E_chitin) were widely separated. By contrast, the three biological replicates of each sorghum line and each MAMP treatment, as well as the mock-treatment, were closely clustered together, respectively, indicating a good reproducibility between biological replicates (Figure 2).

**Figure 3:**
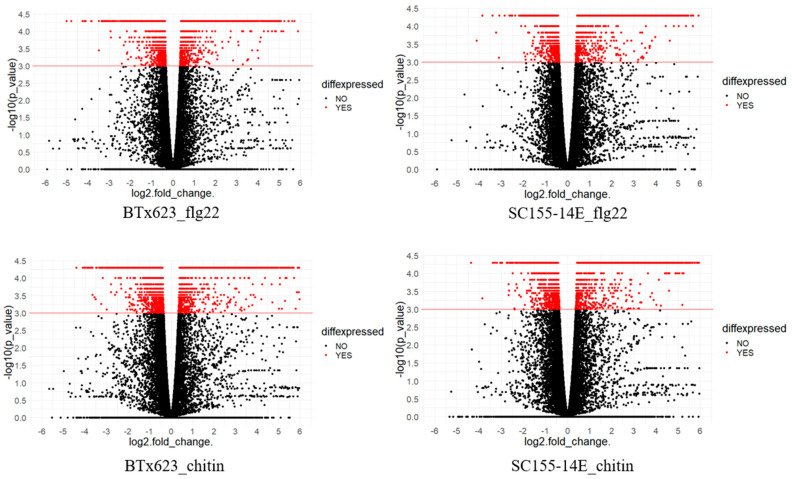
Volcano plots showing differential expression of genes in BTx623_treated with flg22 or chitin and SC155-14E treated with flig22 or chitin. The *x*-axis shows log2fold-changes in expression and *y*-axis shows statistical significance (−log10 of the *p*-value). The red color dots represent DEGs and the black color dots represent the non-DEGs as marked in figure. Down-regulated transcripts are plotted on the left, up-regulated transcripts on the right.

Volcano plots in Figure 3 show gene expression in the two sorghum lines treated with flg22 or chitin. A volcano plot is a type of scatterplot that shows statistical significance (*p*-value) versus magnitude of change (fold change). It enables quick visual identification of genes with large fold changes that are also statistically significant. These may be the most biologically significant genes. In Figure 3, genes with −log10 (*p*-value) equal or greater than 3 are considered as DEGs. The red color dots represent DEGs and the black color dots represent the non-DEGs. In each plot, genes with log2 (fold_change) greater than 0 are up-regulated genes, otherwise, down-regulated genes. The most up-regulated genes are towards the right, the most down-regulated genes are towards the left, and the most statistically significant genes are towards the top.

Differentially expressed genes (DEGs) were selected from each treatment according to their significance of expression (*p*-value 0.001). It should be noted that we used a *p*-value of 0.001 as our threshold but not FDR (*q*-values) to select DEGs in this report. The FDR (*q*-values) were also significant at this level (Tables S2–S5). At a *p*-value of 0.001, the FDRs were 0.0039, 0.0051, 0.0025 and 0.0035 for BTx623 with flg22 treatment, SC155-14E with flg22 treatment, BTx623 with chitin treatment and SC155-14E with chitin treatment, respectively. The *p*-values and *q*-values of the genes of BTx623 and SC155-14E in all conditions are listed in Tables S2–S5. By analyzing the expression patterns of these genes in MAMP-treated leaves of BTx623 and SC155-14E, we observed a large number of genes (14–30% of the total expressed genes) that exhibited big differences after flg22 or chitin treatment in both sorghum lines. The number of total DEGs of chitin treated BTx623 (8085) was very close to that reported in a previous transcriptome study in BTx623 infected by the anthracnose pathogen *C. sublineola* (8078) [37]. The numbers of DEGs were higher in flg22 (19%) or chitin (30%) treated BTx623 leaves relative to SC155-14E (14% and 21%, respectively). Although the higher number of DEGs in the MAMP-treated BTx623 relative to SC155-14E was opposite to the ROS response of these two genotypes, this is perhaps not surprising since the ROS response and gene expression response to MAMP treatment occur at different times, are largely independent, and are mediated by different mechanisms [42,43]. The GO term and KEGG pathway enrichment revealed more immunity and pathogen resistance related DEGs presented in MAMP treated SC155-14E samples. Moreover, the fact that numbers of DEG were higher in the chitin-treated samples relative to those treated with flg22 matched well with ROS response assays (Figure 1B) where we saw a stronger ROS response upon chitin treatment.

**Figure 4:**
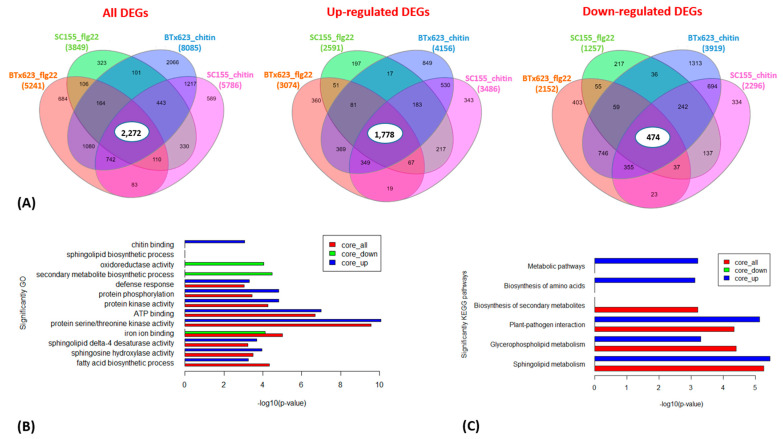
(**A**) Venn diagrams showing the numbers of overlapping and non-overlapping MAMP-responsive genes between BTx623 and SC155-14E at *p* = 0.001. The total, up-regulated and down-regulated DEGs and the treatments are indicated. The numbers of total, up- and down-regulated core DEGs are highlighted in the middle. (**B**) Enriched gene ontology (GO) terms associated with core sets of total, up- and down-regulated DEGs in flg22 or chitin treated BTx623 and SC155-14E were shown with *p*-values indicated on x-axes. (**C**) Enriched KEGG pathways of the total, up- and down-regulated core DEGs in flg22 or chitin treated BTx623 and SC155-14E with *p*-values indicated on x-axes.

To investigate to what extent genes and biological processes are shared between the flg22 and chitin treatments in BTx623 and SC155-14E, we compared their DEGs in more detail. Venn diagrams in Figure 4A show the overlap in DEGs in the total, the up- and down-regulated gene sets at a *p*-value of 0.001. Out of the total of 10,535 DEGs, a core set of 2272 genes responded to both flg22 and chitin treatment in both sorghum lines. Similarly, from the total of 5410 up-regulated genes, a core set of 1778 genes were up-regulated in both genotypes, while, out of a total of 5125 down-regulated genes, a core set of 474 were down-regulated in both genotypes.

**Figure 5:**
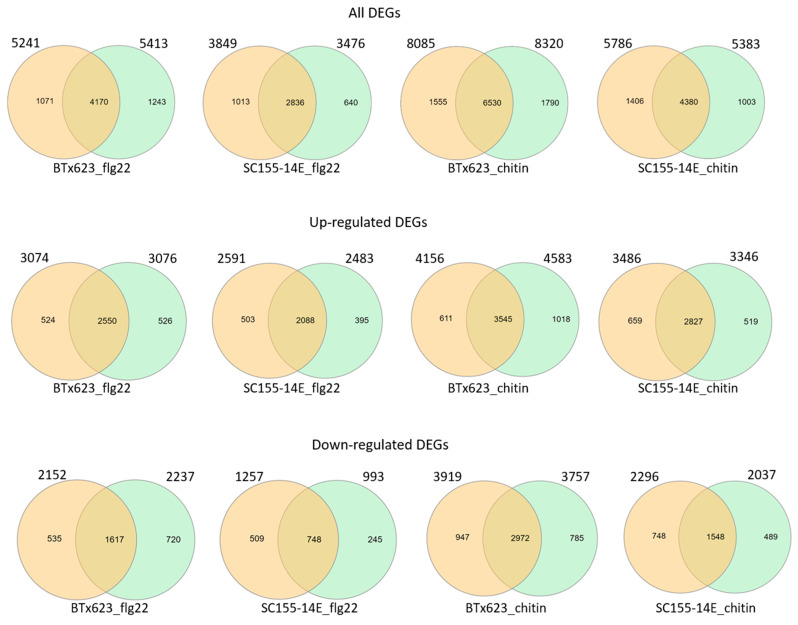
Venn diagrams showing the pairwise overlap of total, up- and down-regulated DEGs selected using pipeline #1 and pipeline #2 of the genotypes and treatments as marked in figures. The circles and numbers on the left of each pair represent DEGs selected using pipeline 1 and the circles and numbers on the right of each pair represent DEGs selected using pipeline 2.

RNA seq data presented here was analyzed using bioinformatics pipeline with tools Bowtie2 2.3.4.3, TopHat 2.1.1, and Cufflinks 2.2.1 (Cuffmerge, Cuffdiff) with default parameters (pipeline #1). To further confirm the assay results, we used another pipeline (pipeline #2) with HiSat2, HTSeq, and edgeR to perform the analysis. HiSat2 is the next development of TopHat2. We compared the DEGs of each condition (i.e., two genotypes treated with flg22 or chitin, all, up- or down-regulated DEGs) selected using both pipelines. The results (Figure 5) showed that the DEG numbers selected using both pipelines were very close. The total DEGs selected with pipeline #1 vs. pipeline #2 were BTx623_flg22: 5241 vs. 5413; SC155-14E_flg22: 3849 vs. 3476; BTx623_chitin: 8085 vs. 8320; SC155-14E_chitin: 5786 vs. 5383. The up-regulated DEGs selected with pipeline #1 vs. pipeline #2 were BTx623_flg22: 3074 vs. 3076; SC155-14E_flg22: 2591 vs. 2483; BTx623_chitin: 4156 vs. 4583; SC155-14E_chitin: 3486 vs. 3346. The down-regulated DEGs selected with pipeline #1 vs. pipeline #2 were BTx623_flg22: 2152 vs. 2237; SC155-14E_flg22: 1257 vs. 993; BTx623_chitin: 3919 vs. 3757; SC155-14E_chitin: 2296 vs. 2037. In addition, the results in Figure 5 also revealed that a high percentage of DEGs selected using the two pipelines overlapped in all conditions. The percentage of overlapped DEG numbers/DEG numbers selected using pipeline#1 were 79.6%, 73.7%. 80.8% and 74.4% of total DEGs of BTx623_flg22, SC155-14E_flg22, BTx623_chitin and SC155-14E_chitin, respectively; 83%, 80.6%, 85.3% and 81.1% of up-regulated DEGs of BTx623_flg22, SC155-14E_flg22, BTx623_chitin and SC155-14E_chitin, respectively; 75.1%, 59.5%, 75.8% and 67.4% of down-regulated DEGs of BTx623_flg22, SC155-14E_flg22, BTx623_chitin and SC155-14E_chitin, respectively. Thus, these comparative results proved that the RNA seq data presented are correct regardless of which of the two methods is used for the analysis.

**Figure 6:**
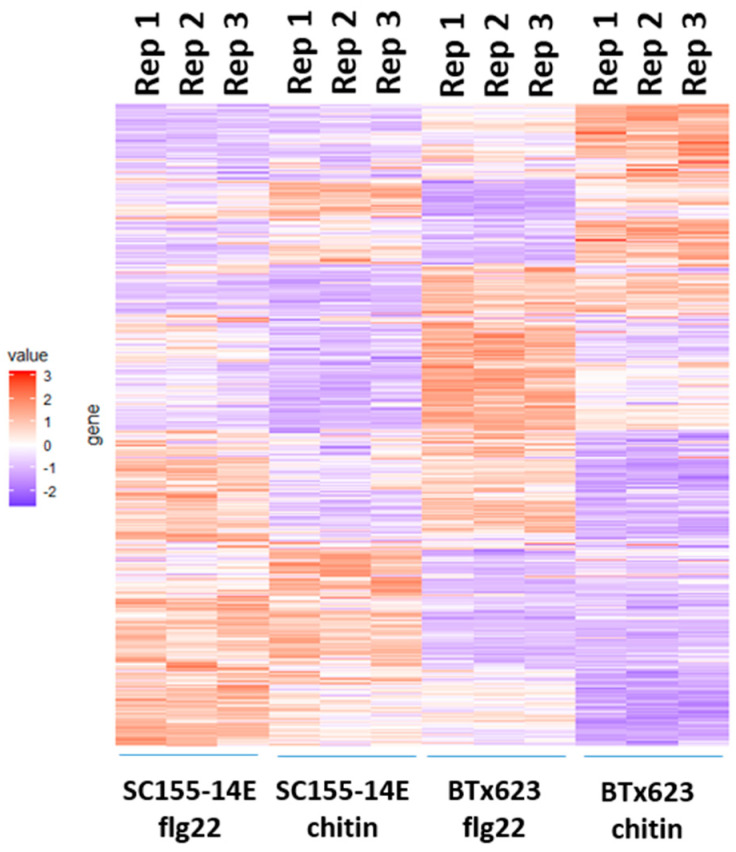
Heat map of transcriptomic response induced by MAMPs in BTx623 and SC155-14E. Three replicates per treatment were analyzed. The gene expression values were normalized as z-scores. The color turns from purple to red as the value increase.

The heatmap of clustered expression profiles of the DEGs of BTx623 and SC155-14E treated with flg22 or chitin for the three biological replicates is shown in Figure 6. The gene expression values were normalized as z-scores. The color turns from purple to red as the value increases, indicating the gene expression from low to high. This heatmap clearly shows the differential regulation of genes in BTx623 and SC155-14E in response to flg22 or chitin treatment. In addition, the results also show the heatmaps of three biological replicates in each condition were very close, indicating a good reproducibility between biological replicates.

**Figure 7:**
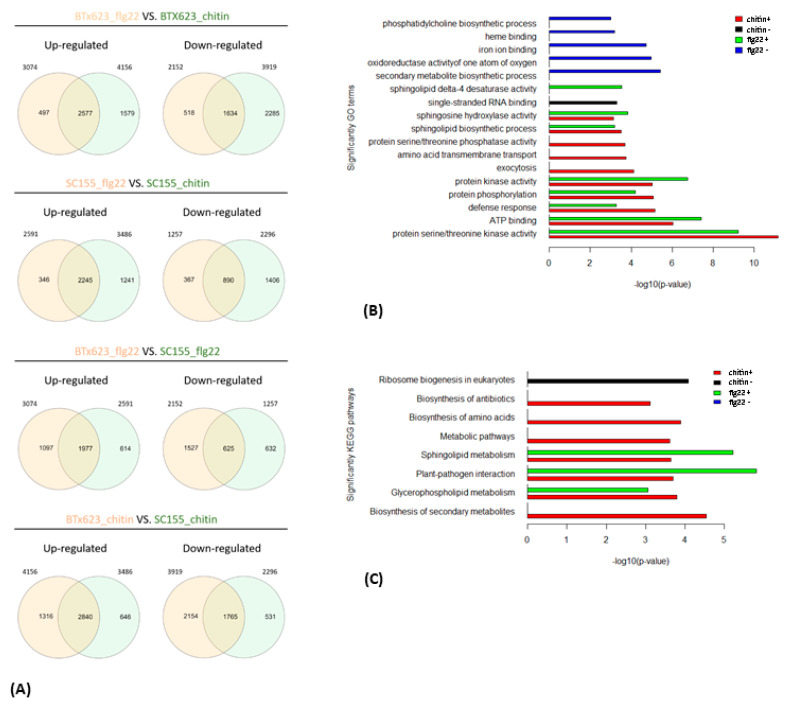
(**A**) Venn diagrams showing the pairwise overlap between the up-regulated and down-regulated genes of the indicated treatments. (**B**) Enriched GO terms of co-regulated genes between the BTx623 and SC155-14E after flg22 or chitin treatment (i.e., up-regulated or down-regulated in both genotypes) with *p*-values indicated on x-axes. (**C**) Enriched KEGG pathways of co-regulated sets of DEGs between the two genotypes after flg22 or chitin treatment with *p*-values indicated on x-axes.

In addition, pair-wise comparisons (Figure 7A) between up- and down-regulated DEGs from the flg22 and chitin treatments showed that there was a large overlap between BTx623 and SC155-14E. Among the 3074 flg22-induced genes in BTx623, 83.8% were also induced in BTx623 in response to chitin treatment, whereas of the 2591 flg22-induced genes in SC155-14E, 86.6% also responded to chitin treated SC155-14E. This is perhaps not surprising since previous works in *Arabidopsis thaliana* showed a significant overlap in genes responding to a variety of distinct MAMPs, which define the general MTI pathways [44,45,46]. Moreover, Zhang et al. [17] also reported a significant correlation between flg22 and chitin response in maize. Less overlap was observed between BTx623 and SC155-14E leaf discs treated with the same MAMP (Figure 7A). 64.3% of flg22-induced genes in BTx623 were induced in flg22-treated SC155-14E and 68.3% chitin-induced genes in BTx623 were induced in chitin treated SC155-14E. Similarly, of the 2152 genes down-regulated upon flg22 treatment of BTx623, a high percentage (75.9%) were also down-regulated by chitin in BTx623. Out of 1257 genes downregulated by flg22 in SC155-14E, 70.8% were also down-regulated by chitin in SC155-14E. However, only 29% of flg22 down-regulated genes in BTx623 were also down-regulated in flg22-treated SC155-14E and 45% of the chitin-down-regulated genes in BTx623 were down-regulated in chitin-treated SC155-14E (Figure 7A). Hence, while there was significant convergence in the MTI pathways to flg22 and chitin in the two genotypes compared, the data also hint at interesting complexity with regard to how these two genotypes respond to different MAMPs.

### 2.3. Gene Functional Enrichment Analysis Comparing the MTI Response of BTx623 and SC155-14E

**Figure 8:**
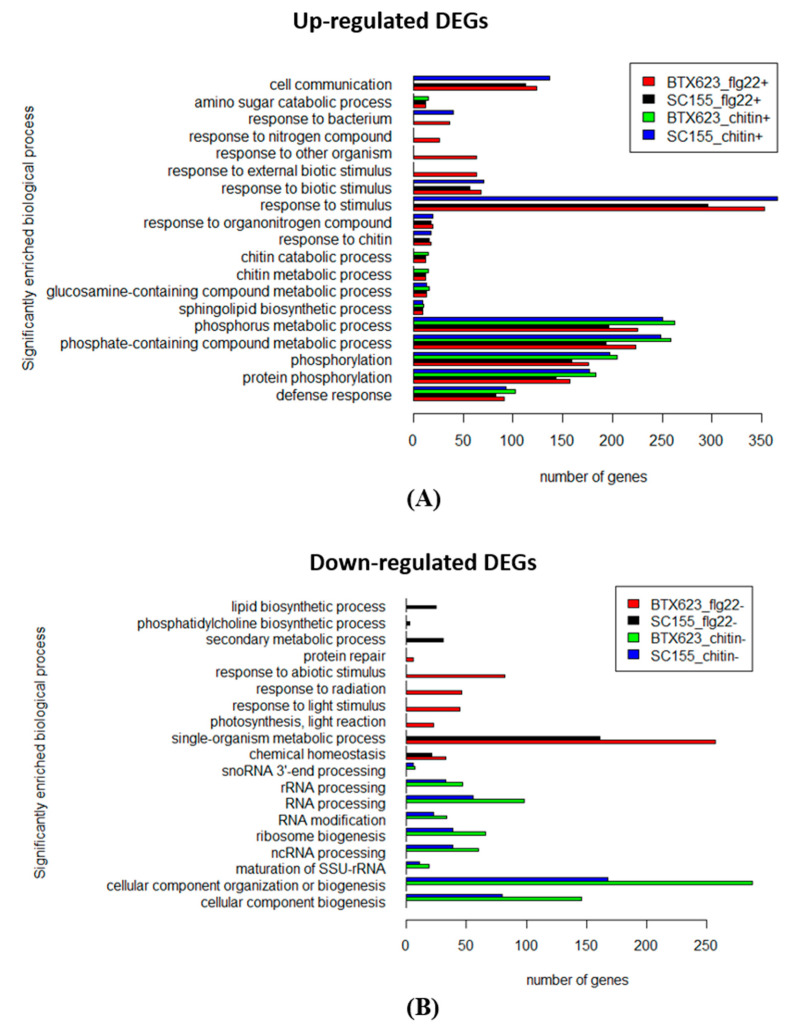
Top GO terms of DEGs in flg22 or chitin treated BTx623 and SC155-14E. Enriched GO terms of detected DEGs were classified into functional categories of biological processes using PlantRegMap platform (**A**) Up-regulated DEGs. (**B**) Down-regulated DEGs.

To determine the functional categories of genes regulated in response to either flg22 or chitin and the differential response of the two sorghum lines tested, we used the PlantRegMap [47,48] platform to perform Gene Set Enrichment Analysis based on the detected DEGs in the biological process category. Figure 8A,B shows the top GO terms of biological process enriched genes with up-regulated DEGs and down-regulated DEGs from both BTx623 and SC155-14E. The complete list of DEGs is provided in Tables S6–S13. Many of these genes are associated with processes including stress response, plant defense responses, response to stimulus, response to biotic stimulus, response to bacterium, response to other organism, cell communication, phosphorus metabolic process, and protein phosphorylation. The results in Figure 8A,B shows clear differences in the expression of genes normally associated with innate immunity between the two sorghum genotypes. In those enriched GO terms, some unique DEGs were found in each condition with overlap. For example, the GO term defense response (GO 0006952): 91 and 84 up-regulated DEGs were enriched in BTx623 or SC155-14E treated with flg22, respectively. Within those DEGs, 24 DEGs were unique in BTx623 and 15 DEGs were only found in SC155-14E, and the others were presented in both sorghum genotypes. Similarly, 102 and 93 up-regulated DEGs were enriched in BTx623 or SC155-14E treated with chitin, respectively. Within those DEGs, 20 DEGs were unique in BTx623 and 14 DEGs were unique in SC155-14E, and the others were presented in both sorghum genotypes.

In the GO term of “response to chitin (GO: 0010200)”, we observed 18 up-regulated DEGs out of a total of 779 genes in this category enriched in the chitin-treated SC155-14E samples (Figure 8), whereas no gene was enriched in the chitin-treated BTx623 samples. In addition, 16 up-regulated DEGs were enriched in flg22-treated SC155-14E samples and 12 up-regulated DEGs enriched in flg22-treated BTx623 samples (Figure 8). Comparison of the DEG lists of GO term “response to chitin” in flg22 and chitin treated BTX623 and SC155-14E (Table S9) revealed that the DEGs enriched in flg22, or chitin treated SC155-14E were the same except chitin induced two more DEGs than flg22. By contrast, all 12 DEGs enriched in flg22 treated BTX623 were different from DEGs induced by MAMPs in SC155-14E. Those MAMPs induced DEGs may contribute to the resistance response of anthracnose in SC155-14E since chitin is a typical MAMP molecule from fungal cell walls which elicits plant immune responses. Stringlis et al. [49] also reported a set of up-regulated DEGs were enriched in GO term of “response to chitin” from chitin, flg22, and a plant beneficial rhizobacteria *Pseudomonas simiae* WCS417 treated *Arabidopsis* plants. Matic et al. [33] reported that 21 DEGs were enriched in the GO term of “response to chitin” from the rice leaf RNA-seq analysis of resistant cultivar but not from a susceptible cultivar after infection with the fungal pathogen *Fusarium fujikuroi*.

PR (Pathogenesis-related) genes are involved in the plant immune response [50] and have antifungal activity against many phytopathogenic fungi [51]. PR-10 proteins are small, primarily acidic, intracellular proteins with antifungal properties that have been associated with defense responses in plant species including sorghum [52]. PR-10 gene family members were induced by pathogen attack in a wide variety of plant species [53]. In sorghum, Lo et al. [26] reported that PR-10 expression was induced as part of the active host defense of sorghum against foliar fungal pathogens *C. heterostrophus* and *C. sublineolum*. Katile et al. [54] reported that several sorghum cultivars showed significant induction of normalized relative quantities of PR-10 after inoculation with fungal spores of *C. lunata* and *F. thapsinum* in field tests. In the greenhouse condition, the glumes of inoculated plants showed induction of PR-10 mRNA, and the response was greater in two resistant cultivars tested relative to two susceptible cultivars. Our results revealed that among the DEGs identified, four PR-10 genes were found (SORBI_3001G401300, SORBI_3001G401200, SORBI_3001G400800, SORBI_3001G401000). In SC155-14E all four PR-10 DEGs were up-regulated by flg22 or chitin treatment, by contrast, in BTx623, SORBI_3001G401000 was not found in DEGs, and SORBI_3001G401300 (up-regulated) was only presented in BTx623 with chitin treatment but not with flg22 treatment. This indicated that expression of PR genes could be employed in response to higher ROS product levels after MAMPs treatment and resistance against anthracnose in SC155-14E.

Salicylic acid (SA) is a plant immune signal essential for both local defense response and systemic acquired resistance. It plays an important role in resistance and plant defense against pathogen attacks [55]. In sorghum, Tugizimana et al. [56] reported quantitative changes in the levels of jasmonic acid, salicylic acid conjugates, and abscisic acid in *C. sublineola* infected sorghum. *C. sublineola* is the causal agent of anthracnose. Sorghum genotypes with enhanced levels of amino acids (tryptophan and tyrosine), jasmonic acid and salicylic conjugates, and zeatin were more resistant to anthracnose. In this study, the GO term ‘response to salicylic acid’ (GO:0009751) including 19 up-regulated DEGs was specifically enriched only in SC155-14E treated with chitin (Table S9). Hence, the salicylic signaling pathway could play an important role in response to higher ROS production levels after MAMP treatment and resistance against anthracnose in SC155-14E.

Furthermore, 169 up-regulated DEGs were enriched in GO term response to stress solely in SC155-14E treated with chitin (Table S9). The other GO terms found solely in SC155-14E treated with MAMPs, but not in BTx623 with the same treatment are secondary metabolic processes (GO:0019748), phosphatidylcholine biosynthetic process (GO:0006656), and lipid biosynthetic process (GO:0008610).

Irrespective of the higher number of DEGs in the MAMP-treated BTx623 relative to SC155-14E, these more refined GO term comparisons are consistent with the fact that SC155-14E plants exhibit a stronger MTI response and resistance to fungal disease anthracnose, relative to BTx623.

We also examined the composition of genes that are co-regulated between the two genotypes after flg22 or chitin treatment as well as the core sets of DEGs in all treatment and two genotypes by GO enrichment analysis (Figure 4B and Figure 7B). The full list of DEGs is provided in Tables S14 and S15. Figure 4B showed the enriched GO terms of the core sets of the total, up- and down-regulated DEGs in BTx623 and SC155-14E with all treatments. The GO terms related to MAMPs response and plant defense (e.g., chitin binding, defense response, protein kinase activity, protein phosphorylation, protein serine/threonine kinase activity) were significantly enriched in up-regulated DEGs while GO terms of secondary metabolite biosynthetic process, iron ion binding, oxidoreductase activity were only enriched in down-regulated DEGs. This result was similar to results in *Arabidopsis* where GO terms related to response to elicitors (flg22 peptides from *P. simiae* WCS417 and *P. aeruginosa* PAO1, chitin, and bacterial cells of *P. simiae* WCS417) were most significantly enriched in the up-regulated core set of DEGs [49]. Figure 7B showed the significantly enriched GO terms of co-regulated (i.e., up- or down-regulated in both genotypes) sets of DEGs between BTx623 and SC155-14E after flg22 or chitin treatment. The results revealed that GO terms related to MAMPs response and plant defense (e.g., defense response, protein kinase activity, protein phosphorylation, protein serine/threonine kinase activity) were enriched only in flg22 or chitin up-regulated DEGs in both genotypes while GO terms of secondary metabolite biosynthetic process, heme binding, iron ion binding, oxidoreductase activity of one atom of oxygen and phosphatidylcholine biosynthetic process were enriched solely in down-regulated sets of DEGs.

### 2.4. KEGG Pathway Enrichment Analysis of the MTI Response of BTx623 and SC155-14E

**Figure 9:**
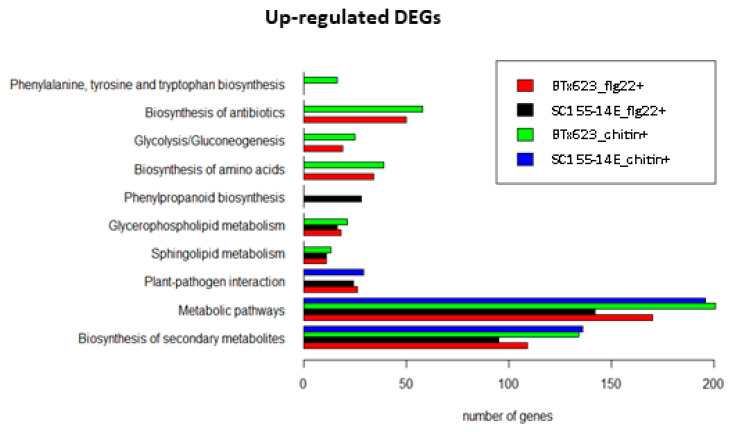
Top KEGG pathways of up-regulated DEGs in flg22 or chitin treated BTx623 and SC155-14E.

Figure 9 shows the KEGG pathway enrichment analysis of the up-regulated DEGs found in the MAMP treated BTx623 and SC155-14E samples using the DAVID online tool [57]. The full list of DEGs is provided in Tables S16–S20. The results identify those specific top KEGG pathways enriched as a result of activation of MTI in the two sorghum genotypes. In the KEGG pathway “plant-pathogen interaction”, we observed that 24 up-regulated DEGs were enriched in the chitin treated SC155-14E samples, while none of the DEGs were enriched in the chitin treated BTx623 samples. The stronger chitin response of genotype SC155-14E does correlate with the greater resistance of this genotype to the fungal disease anthracnose [39]. Moreover, KEGG pathway enrichment revealed 28 genes involved in term phenylpropanoid biosynthesis in SC155-14E treated with flg22. The phenylpropanoid biosynthesis pathway begins with phenylalanine which can be converted into aromatic compounds such as flavonoids, benzenoids, coumarine, hydroxycinnamates, and lignin [58,59]. Many phenylpropanoids and flavonoids were involved in the disease resistance response [28,60]. Production of phytoalexins is a major defense response against anthracnose pathogen *C. sublineola* [28] in sorghum. The 3-deoxyanthocyanidins are an unusual group of flavonoids recognized as phytoalexins in sorghum. Phytoalexins are small molecules with antimicrobial activity produced after pathogen infection [61]. Wang et al. [37] also found that KEGG term phenylpropanoid biosynthesis was significantly enriched in DEGs of *C. sublineola* infected plants in their transcriptomics study of sorghum. We should note that KEGG pathway enrichment results (Figure 9) also showed a higher response of BTx623 after MAMPs treatment in some pathways which might play a role in plant-pathogen interactions (e.g., in pathways phenylalanine and tryptophan biosynthesis, antibiotic biosynthesis). Furthermore, results of KEGG pathway enrichment analysis of the down-regulated DEGs revealed that ten down-regulated DEGs were annotated as ‘photosynthesis-antenna proteins’ and 84 DEGs were annotated as being participated in the ‘biosynthesis of secondary metabolites’ were enriched in flg22 treated BTx623. However, such gene expression studies can identify genes differentially expressed in response to MAMPs treatment or pathogen infection, but it does not necessarily demonstrate that these genes are critical for resistance against a specific pathogen. Thus, further research would be needed to understand the causative relationships between fungal resistance and the activation of those KEGG pathways in both genotypes, as revealed by RNA-seq analysis.

We also performed KEGG pathway enrichment analysis for the core sets of DEGs in all conditions (Figure 4C) as well as the co-regulated DEGs between the two genotypes after flg22 or chitin treatment (Figure 7C). The results revealed that six significant pathways, including the plant-pathogen interaction pathway, were enriched only in up-regulated core sets of DEGs but not in down-regulated core sets of DEGs. Similarly, seven and three pathways, including the plant-pathogen interaction pathway, were significantly enriched in chitin up-regulated DEGs and flg22 up-regulated DEGs in BTx623 and SC155-14E, respectively. By contrast, only ribosome biogenesis in eukaryotes pathway was enriched in chitin down-regulated DEGs in both genotypes.

### 2.5. Validation of the RNA-Seq Data Using Quantitative RT-PCR of Select Genes

To confirm the gene expression profiling data obtained by RNA-seq, we performed qRT-PCR analysis to evaluate the expression of nine selected candidate genes. Those nine genes were selected based on the expression observed by RNA-seq in three categories: (a) expressed similarly in BTx623 and SC155-14E with flg22 or chitin treatment; (b) expressed only in MAMPs treated SC155-14E but with extremely low expression in treated BTx623 and (c) expressed 2–21-fold higher in MAMPs treated SC155-14E relative to BTx623. The qRT-PCR analysis results (Table 1) revealed a full agreement with the expression levels determined by RNA-seq analysis for each of the nine genes evaluated. These results give us confidence that the measurements made by RNA-seq are reflective of the transcriptional response of the sorghum lines to MAMP treatment.

### 2.6. Conclusions

In this study, we first found the optimal conditions for comparison of the MTI response of the two genotypes by testing ROS production using three sets of leaves and chose the second youngest leaves of fifteen-day-old plants for RNA extraction and subsequent transcriptomic analysis. A time course of flg22-triggered and chitin-triggered ROS production in sorghum line SC155-14E (Figure 1C) revealed that chitin-triggered ROS production occurred earlier than that triggered by flg22 treatment. We obtained the expression profiles of the fungal disease anthracnose resistant *Sorghum bicolor* genotype SC155-14E with high response to MAMPs treatment on ROS production and the susceptible genotype BTx623 with a low response on ROS product to MAMP treatment during the early stages of the innate immunity response. The results demonstrate a clear variation of gene expression in the sorghum genotypes BTx623 and SC155-14E in response to MAMP treatment. While the responses to the two MAMPs showed considerable overlap within each line (86.6–70.8%), they were clearly distinct. Some, albeit lower, overlap (68.3–29%) was also observed between the responses of the two lines to the same MAMP. A considerable number of DEGs, 2272 out of 10,535 DEGs, were identified in all four conditions and tentatively define the core MAMP response. This list of genes should be useful to those laboratories wanting to profile the gene expression response to MAMP treatment. The RNA-seq analysis identified large sets of differentially expressed genes in BTx623 and SC155-14E treated with flg22 or chitin compared to untreated mock controls of those two lines. Furthermore, a comparison of DEGs selected using two different bioinformatics’ pipelines showed very similar results. Detailed expression profile analysis of these DEGs, GO enrichment analysis and KEGG pathway analysis revealed a clear variation in the gene expression response of the two genotypes. The GO term and KEGG pathway enrichment discovered more immunity and pathogen resistance related DEGs (PR genes, genes response to chitin, genes response to salicylic acid, response to stress, phenylpropanoid biosynthesis, etc.) in MAMP treated SC155-14E samples relative to BTx623 with the same treatment. This information provides important baseline information on how the innate immune system of this key crop plant responds to MAMPs elicitation. Given the general lack of information on the innate immune response of crop plants, relative to the model *Arabidopsis*, the gene lists and methods described provide a resource for further exploration of the response of sorghum to pathogens and should facilitate efforts to ultimately improve disease resistance in this important food and biomass crop.

## 3. Materials and Methods

### 3.1. Plant Materials

Two sorghum lines provided by Dr. William Rooney (Texas A&M University, College Station, USA) and Dr. Stephen Kresovich (Clemson University, Clemson, USA) were used in this study. BTx623 is a standard sorghum line with an available whole genome sequence [22], and SC155-14E is a line developed for anthracnose resistance [39].

### 3.2. ROS Assays 

Sorghum seeds of BTx623 and SC155-14E were surface sterilized (70% ethanol for 1 min and then 10% bleach for 10 min, rinsed with autoclaved ddH_2_O) and planted in autoclaved Sunshine potting mix, and germinated in growth chambers (16 h/8 h light/dark, 28/26 °C, 60–70% humidity). Fifteen-day-old plants were used for experiments.

Two MAMPs, flg22 (Genscript catalog# RP19986) and chitin from crab shell (Sigma-Aldrich, catalog# C3641), were used in this study. Flg22 is a peptide derived from the flagellin N-terminus of *plant pathogenic bacteria* and is well known to elicit a specific innate immune response in plants [62]. Chitin is a typical MAMP molecule derived from fungal cell walls, which elicits plant immune responses [3]. ROS assays were performed according to Kimball et al. [38]. To assess the variation in the MAMP response within individual genotypes, as well as the position of leaves, all three fully expanded leaves of the plants of each genotype were assessed individually. The leaves from the bottom to the top of the fifteen-day-old sorghum plants of each genotype were marked as leaf #1, #2, and #3. We used only the middle part of the leaf for sampling to measure ROS production. Immediately after treatments, the chemiluminescent signal of each sample was recorded for 30 min using a Photek CCD camera (Photek Ltd., East Sussex UK). Eight wells consisted of a mock treatment (without MAMP) and eight wells consisted of treatment (with MAMPs). In every case, three biological replicates with a total of 8 samples were compared for each treatment.

### 3.3. Sample Treatment with MAMPs

Fifteen-day-old plants were used for experiments. The MAMPs treatments were performed as described in Valdes-Lopez et al. [12] with minor modification. Briefly, the second leaf (from top- i.e., the second youngest leaf) from five plants for each sorghum line were detached and then vacuum infiltrated with autoclaved ddH_2_O for 2 min. About fifty 1 cm diameter leaf discs were cut from the water-infiltrated leaves of each genotype and pooled. Forty-five leaf discs from each genotype were transferred into three different petri dishes (15 leaf discs each petri dish) and then floated overnight at room temperature on autoclaved ddH_2_O with the plates covered with aluminum foil. The next day, the water was removed from all petri dishes and replaced with 10 mL of ddH_2_O (mock), 10 mL 1 µM flg22, or 10 mL of 20 mg/mL chitin solution. After a 60-min treatment, mock- and MAMP-treated leaf slices were harvested into different tubes and immediately frozen in liquid nitrogen. The leaf slice samples (for 101quenced “ies ied-11272) immunityBTx623 and SC155-14E with mock, flg22, or chitin treatments, three biological replicates) were stored at −80 °C for RNA extraction. All procedures described above were performed under dark conditions to eliminate any possible photosynthesis effect.

### 3.4. RNA Extraction, Sequencing and Library Construction

The RNA extraction was performed using the Direct-zol RNA Miniprep Plus kit from Zymoresearch (catalog #R2071) according to the manufacturer’s instructions.

High-throughput sequencing was performed at the University of Missouri DNA Core Facility. Eighteen libraries were constructed following the manufacturer’s protocol with reagents supplied in the Illumina’s TruSeq mRNA stranded sample preparation kit. The sample concentration was determined by Qubit fluorometer (Invitrogen) using the Qubit HS RNA assay kit, and the RNA integrity was checked using the Fragment Analyzer automated electrophoresis system. Briefly, the poly-A containing mRNA was purified from total RNA (1 µg), RNA was fragmented, double-stranded cDNA generated from fragmented RNA, and the index containing adapters ligated to the ends. The amplified cDNA constructs were purified by the addition of Axyprep Mag PCR Clean-up beads. The final construct of each purified library was evaluated using the Fragment Analyzer automated electrophoresis system, quantified with the Qubit flourometer using the Qubit HS dsDNA assay kit, and diluted according to Illumina’s standard sequencing protocol for sequencing on the NextSeq 500. The sequencing length was single read at 75 bases.

### 3.5. Mapping and Processing of RNA-Seq Reads

The sequence data represented six different conditions: BTx623 with mock, flg22 or chitin treatment, and SC155-14E with mock, flg22 or chitin treatment. Each condition was represented by three biological replicates, resulting in 18 total samples. First, the 3′ end of the reads were “trimmed” for Illumina adapters, for ambiguous nucleotides (N’s), and (because of NextSeq technology) for artificial poly-G (represented as G{100}) using cutadapt version 1.15 (http://dx.doi.org/10.14806/ej.17.1.200, accessed on 21 August 2019) for reads whose 3′ ends overlap with the adapter for a minimum of 3 bases with 90% identity. If after this trimming, a read contained fewer than ten bases it was discarded (along with its paired read, if applicable). The quality scores for the RNA seq data after trimming were examined using FASTQC (version 0.11.9) [63]. For all the positions in reads, the median quality score is around 34, indicating the base calling accuracy is higher than 99.9%. Reads for each sample were aligned to the reference genome (Sorghum_bicolor_NCBIv3.dna.toplevel.fa) with Tophat 2.1.1. The resulting alignment files were provided to Cufflinks 2.2.1 to assemble transcripts for each sample. The annotation version used in this research was Sorghum_bicolor_NCBIv3.38.gff3. Samples from the same condition were merged together using Cuffmerge. The purpose was to provide a uniform basis for calculating gene and transcript expression in each condition.

### 3.6. Bioinformatic Analysis of RNA-Seq Data

RNA seq data was analyzed using bioinformatics tools Bowtie2 2.3.4.3, TopHat 2.1.1, and Cufflinks 2.2.1 (Cuffmerge, Cuffdiff) with default parameters. We used the same pipeline with these tools as described in Trapnell et al. [64]. To further confirm the assay results, we used another pipeline with HiSat2 (version 2.1.0), HTSeq (version 0.12.4), and edgeR (version 3.26.8) to perform the analysis. HiSat2 is the next development of TopHat2. First, we used HiSat2 to do the alignment using the Sorghum reference genome. Then we used HTSeq to do the counting of how many reads that mapped to each gene. For this, we needed the bam files generated from the previous step and the genome annotation (gtf file). Note that the reference genome and the annotation files are the same as we used in the original pipeline. Finally, we used the R package edgeR to determine the differentially expressed genes in each condition.

### 3.7. Identification of Differentially Expressed Genes

The merged assembly was fed to Cuffdiff, which calculates expression levels and tests the statistical significance of observed changes between two conditions. We compared the conditions between mock vs. flg22, mock vs. chitin for BTx623, and SC155-14E, respectively. The differentially expressed genes were extracted using 0.001 *p*-value thresholds. The volcano plots were made using the R package (ggplot2 V3.3.0) to show the significance of gene expression change in conditions BTx623 treated with flg22 or chitin and SC155-14E treated with flg22 or chitin, respectively. The gene dots with −log10 (*p*-value) equal or greater than three were considered as DEG. In each plot, the gene dots with log2 (fold_change) greater than zero were up-regulated genes, otherwise, down-regulated genes.

### 3.8. PCA Plot

To examine the relationship between gene expression values of three biological replicates of each sorghum line and each MAMP treatment, principal component analysis (PCA) plots were generated using R packages (ggfortify V0.4.10, ggplot2 V3.3.0). Each dot represents a condition after mapping from the original feature space (gene expression) to the first two principle components. Dots belonging to the same condition tend to cluster together.

### 3.9. Heat Map of DEGs 

Heat map of the transcriptomic response induced by MAMPs in BTx623 and SC155-14E was constructed using ggplot2 (V3.3.0), ggdendro (V0.1.21), reshape2 (V1.4.4).

### 3.10. Gene Ontology Enrichment

All the DEGs were divided into eight groups, up-regulated genes in BTx623 or SC155-14E treated with flg22 or chitin, and down-regulated genes in these conditions. Each DEG group was fed to the GO enrichment tool provided by PlantRegMap [47,48] to perform Gene Set Enrichment Analysis in the biological process category. The threshold *p*-value was set as 0.001 and we selected the terms with an FDR < 0.01 in at least one of the four conditions (i.e., BTx623/SC155-14E treated with flg22 or chitin).

### 3.11. KEGG Pathway Enrichment

The eight groups of differentially expressed genes were fed to the DAVID online tool [57] to do a KEGG pathway enrichment analysis. We used Benjamini–Hochberg procedure to control the FDR at level 0.01.

### 3.12. GEO Accession Number

The RNA-seq data and analysis results were deposited in NCBI’s Gene Expression Omnibus (GEO) and are accessible through the GEO accession number, GSE151860 (link: https://www.ncbi.nlm.nih.gov/geo/query/acc.cgi?acc=GSE151860, accessed on 10 December 2020).

### 3.13. qRT-PCR to Validate RNA Sequence Data

To verify the accuracy of the RNA-seq data, we performed quantitative reverse transcription PCR (qRT-PCR) to evaluate the expression of nine selected genes. qRT-PCR was performed as described in Libault et al. [65,66] in an Applied Biosystem qPCR machine (95 °C 10 min, and 45 cycles of 95 °C 10 s, 60 °C 1 min). SORBI_3004G039400 (*EIF4*α) was used as a reference gene to normalize the expression levels of the analyzed genes [67]. Primer design was performed using NCBI Primer-blast (https://www.ncbi.nlm.nih.gov/tools/primer-blast, accessed on 10 December 2019). The primers used for qRT-PCR were listed in Table S1. Expression levels of the analyzed genes were calculated according to the equation E = P_eff_^(−ΔCt)^, where P_eff_ is the primer set efficiency calculated using LinRegPCR [68]. The ΔCt was calculated by subtracting the cycle threshold (Ct) value of the reference gene from the Ct values of the gene analyzed. Fold changes were calculated between the ratios of the expression levels of MAMP-treated and mock samples, and expression levels were calculated for three biological replicates.

## Figures and Tables

**Table 1:** Comparison of expression levels detected by RNA-seq and q-RT PCR of selected genes with flg22 or chitin treatment.

Gene	Annotation	Expression Ratio of flg22 Treated (RNA-seq)	Expression Ratio of flg22 Treated (qRT-PCR)	Expression Ratio of Chitin Treated (RNA-seq)	Expression Ratio of Chitin Treated (qRT-PCR)	Category
		SC155-14E/BTx623	SC155-14E/BTx623	SC155-14E/BTx623	SC155-14E/BTx623	
SORBI_3006G217900	integral component of membrane [GO:0016021]; ATP binding [GO:0005524]; protein kinase activity [GO:0004672]	0.925	primer set 1: 0.87 primer set 2: 0.79	1.13	primer set 1: 1.24 primer set 2: 1.36	a
SORBI_3002G260900	integral component of membrane [GO:0016021]; ATP binding [GO:0005524]; protein kinase activity [GO:0004672]	0.9	primer set 1:1.39 primer set 2: 1.22	1.04	primer set 1: 0.99 primer set 2: 0.91	a
SORBI_3004G052500	hypothetical protein	29,666	primer set 1: 50,000 primer set 2:60,000	30,000	primer set 1: 40,000 primer set 2: 40,000	b
SORBI_3006G261500	oxidoreductase activity [GO:0016491]	15.3	primer set 1: 20.8 Primer set 2: 19.6	16.6	primer set 1: 13.5 Primer set 2: 13.6	c
SORBI_3007G120401	hypothetical protein	3.64	primer set 1: 2.3 Primer set 2: 2.8	2.72	primer set 1: 2.4 primer set 2: 2.1	c
SORBI_3007G074200	hypothetical protein	1.44	Primer set 1: 1.7 primer set 2: 1.65	1.96	primer set 1: 1.36 Primer set 2: 1.43	a
SORBI_3008G191300	ATP binding [GO:0005524]	2.4	primer set 1: 2.8 primer set 2: 3.0	4.15	primer set 1: 2.8 primer set 2: 2.7	c
SORBI_3010G117800	hypothetical protein	2.51	primer set 1: 1.8 Primer set 2: 1.5	2.79	primer set 1: 1.54 Primer set 2: 2.2	c
SORBI_3003G036200	chloroplast [GO:0009507]; malate dehydrogenase (decarboxylating) (NAD+) activity [GO:0004471]; malate dehydrogenase (decarboxylating) (NADP+) activity [GO:0004473]; metal ion binding [GO:0046872]; NAD binding [GO:0051287]; malate metabolic process [GO:0006108]; pyruvate metabolic process [GO:0006090]	1.06	primer set 1: 1.38 primer set 2: 1.2	1.02	primer set 1:1.2 primer set 2: 1.1	a

Note: Two set of primers were used for qRT-PCR analysis. The sequences of the primers were listed in Table S1. The genes were divided into three categories based on their expression criteria: a expressed similarly in BTx623 and SC155-14E with flg22 or chitin treatment; b expressed only in MAMPs treated SC155-14E but with extremely low expression in treated BTx623 and c expressed 2–21-fold higher in MAMPs treated SC155-14E relative to BTx623.

## Data Availability

The RNA-seq data and analysis results were deposited in NCBI’s Gene Expression Omnibus (GEO) and are accessible through the GEO accession number, GSE151860 (link: https://www.ncbi.nlm.nih.gov/geo/query/acc.cgi?acc=GSE151860, accessed on 10 December 2020).

## Supplementary Materials

The following are available online at https://www.mdpi.com/article/10.3390/plants10081536/s1. Table S1. qRT-PCR primers. Table S2. DEGs of BTx623 treated with flg22. Table S3. DEGs of BTx623 treated with chitin. Table S4. DEGs of SC155-14E treated with flg22. Table S5. DEGs of SC155-14E treated with chitin. Table S6. GO pathway enriched in up-regulated DEGs of BTx623 treated with flg22. Table S7. GO pathway enriched in up-regulated DEGs of SC155-14E treated with flg22. Table S8. GO pathway enriched in up-regulated DEGs of BTx623 treated with chitin. Table S9. GO pathway enriched in up-regulated DEGs of SC155-14E treated with chitin. Table S10. GO pathway enriched in down-regulated DEGs of BTx623 treated with flg22. Table S11. GO pathway enriched in down-regulated DEGs of SC155-14E treated with flg22. Table S12. GO pathway enriched in down-regulated DEGs of BTx623 treated with chitin. Table S13. GO pathway enriched in down-regulated DEGs of SC155-14E treated with chitin. Table S14. GO and KEGG pathway enriched in core set of DEGs of BTx623 and SC155-14E treated with MAMPs. Table S15. GO and KEGG pathway enriched in co-regulated DEGs between the two genotypes after flg22 or chitin treatment. Table S16. KEGG pathway enriched in up-regulated DEGs of BTx623 treated with flg22. Table S17. KEGG pathway enriched in up-regulated DEGs of SC155-14E treated with flg22. Table S18. KEGG pathway enriched in up-regulated DEGs of BTx623 treated with chitin. Table S19. KEGG pathway enriched in up-regulated DEGs of SC155-14E treated with chitin. Table S20. KEGG pathway enriched in down-regulated DEGs of BTx623 treated with flg22.
