# Supplementary material for: Variation in Gene Expression between Two Sorghum bicolor Lines Differing in Innate Immunity Response
Source: Plants (Basel). 2021 Jul 27;10(8):1536. doi: 10.3390/plants10081536 (PMC8399927; doi:10.3390/plants10081536)
Supplement: Supplementary file 1 [file plants-10-01536-s001.zip › Table S1 qRT-PCR primers.pdf]

Table S1. qRT-PCR primers used in this study

Name	Gene	Sequence (5'-3')	Primer set
SORBI_3006G217900_F1	SORBI_3006G217900	GCAAGTTCTTGGCACTCCTG	Primer set 1
SORBI_3006G217900_R1	SORBI_3006G217900	ACCGTCCTCATGTACGCAAA	Primer set 1
SORBI_3006G217900_F2	SORBI_3006G217900	TGGCACTCCTGCTAAAAACG	Primer set 2
SORBI_3006G217900_R2	SORBI_3006G217900	CACCGTCCTCATGTACGCAA	Primer set 2
SORBI_3002G260900_F1	SORBI_3002G260900	CGCTGGGAAGATCGATGTCA	Primer set 1
SORBI_3002G260900_R1	SORBI_3002G260900	TTCCCAGAGCGGATAGGTGA	Primer set 1
SORBI_3002G260900_F2	SORBI_3002G260900	ATTGATCCAAGGCTAGGCGG	Primer set 2
SORBI_3002G260900_R2	SORBI_3002G260900	AGGTCTCATCCTGGGCTCTT	Primer set 2
SORBI_3004G052500_F1	SORBI_3004G052500	CTGGTGCAGCTACATTTGCC	Primer set 1
SORBI_3004G052500_R1	SORBI_3004G052500	TCCTTCAGTACCGAACGCAC	Primer set 1
SORBI_3004G052500_F2	SORBI_3004G052500	CGATGACGGGAGCCAAGATT	Primer set 2
SORBI_3004G052500_R2	SORBI_3004G052500	GAGTGCAAACCCAGCCATTG	Primer set 2
SORBI_3006G261500_F1	SORBI_3006G261500	AGACGGTGAAAGTGCGTGAT	Primer set 1
SORBI_3006G261500_R1	SORBI_3006G261500	TAGATGGGGGTGCCTCTCAA	Primer set 1
SORBI_3006G261500_F2	SORBI_3006G261500	ATTGTCATCGGTGGTGGCAT	Primer set 2
SORBI_3006G261500_R2	SORBI_3006G261500	TATGAACACGCCCGCCAATA	Primer set 2
SORBI_3007G120401_F1	SORBI_3007G120401	TCCGGAGGAAGACATGGGTC	Primer set 1
SORBI_3007G120401_R1	SORBI_3007G120401	CGGAGTGACAGATACCCAGC	Primer set 1
SORBI_3007G120401_F2	SORBI_3007G120401	GGGCAAACCCTTCTCTTCTCA	Primer set 2
SORBI_3007G120401_R2	SORBI_3007G120401	AGTGCTTGACCCATGTCTTCC	Primer set 2
SORBI_3007G074200_F1	SORBI_3007G074200	TCCATCCACCCACAGTTTGC	Primer set 1
SORBI_3007G074200_R1	SORBI_3007G074200	GTAGCCTGCTTGGTAGCTCA	Primer set 1
SORBI_3007G074200_F2	SORBI_3007G074200	TCCATCCATCCACCCACAGT	Primer set 2
SORBI_3007G074200_R2	SORBI_3007G074200	GTAGCCTGCTTGGTAGCTCAT	Primer set 2
SORBI_3008G191300_F1	SORBI_3008G191300	AACTTCATCGACGGGCTCTG	Primer set 1
SORBI_3008G191300_R1	SORBI_3008G191300	ACATCTCGATGTGCCGATCC	Primer set 1
SORBI_3008G191300_F2	SORBI_3008G191300	CAACTTCATCGACGGGCTCT	Primer set 2
SORBI_3008G191300_R2	SORBI_3008G191300	GGACATCTCGATGTGCCGAT	Primer set 2
SORBI_3010G117800_F1	SORBI_3010G117800	AGGTGACTGCGTTCGCTGA	Primer set 1
SORBI_3010G117800_R1	SORBI_3010G117800	TCGACAACGCTAAGCCAGA	Primer set 1
SORBI_3010G117800_F2	SORBI_3010G117800	AACCGAGAGACGGGGTTC	Primer set 2
SORBI_3010G117800_R2	SORBI_3010G117800	AGTCACCTCAGAGGCATAGG	Primer set 2
SORBI_3003G036200_F1	SORBI_3003G036200	CAGCACCTACAGGAAGACCG	Primer set 1
SORBI_3003G036200_R1	SORBI_3003G036200	TGTAACCGCTTGCGACAGAG	Primer set 1
SORBI_3003G036200_F2	SORBI_3003G036200	CTACAGGAAGACCGTGCGGAG	Primer set 2
SORBI_3003G036200_R2	SORBI_3003G036200	CCGCTTGCGACAGAGGTG	Primer set 2
SORBI_3004G039400-F	SORBI_3004G039400 (*EIF4α*)	TCCTGTCGCTGTGTACCTTCT	reference gene primer 1
SORBI_3004G039400-R	SORBI_3004G039400 (*EIF4α*)	GATGGGAGGTATGTAGCATCG	reference gene primer 2
